# Subcortical microstructural impairment in amyotrophic lateral sclerosis: clinical correlates of neurite orientation dispersion and density imaging (NODDI) changes

**DOI:** 10.3389/fnins.2026.1757470

**Published:** 2026-02-13

**Authors:** Minoo Sharbafshaaer, Maria Agnese Pirozzi, Giuseppina Caiazzo, Fabrizio Canale, Marcello Silvestro, Antonio Russo, Alessandro Tessitore, Fabrizio Esposito, Francesca Trojsi

**Affiliations:** 1 Neurology Unit, First Division of Neurology and Neurophysiopathology, AOU University of Campania “Luigi Vanvitelli”, Naples, Italy; 2 Department of Advanced Medical and Surgical Sciences (DAMSS), MRI Research Center, University of Campania “Luigi Vanvitelli”, Naples, Italy

**Keywords:** amyotrophic lateral sclerosis, clinical disability, deep gray matter, NODDI, subcortical nuclei

## Abstract

**Introduction:**

Amyotrophic lateral sclerosis (ALS) is a progressive neurodegenerative disease involving widespread network disruption beyond the motor cortex. Deep gray matter (DGM) nuclei, crucial for motor and cognitive integration, remain underexplored *in vivo*. This study applied neurite orientation dispersion and density imaging (NODDI) to evaluate DGM microstructure and its relationship with clinical disability in ALS.

**Methods:**

Diffusion-weighted MRI data were acquired from 23 ALS patients and 24 age- and sex-matched healthy controls. Orientation dispersion index (ODI), neurite density index (NDI), and free water fraction (FWF) were extracted from the bilateral thalamus, caudate, putamen, pallidum, hippocampus, and amygdala using the Destrieux atlas. Group comparisons and partial correlations were adjusted for age, sex, and disease duration.

**Results:**

No significant group differences in DGM volumes or NODDI-derived metrics survived correction for multiple comparisons. Within the ALS group, several nominal (uncorrected) associations were observed between DGM microstructural metrics and ALSFRS-R subscores. Reduced respiratory subscores were associated with higher ODI in the left thalamus (ρ = 0.57, *p* = 0.0047, uncorrected). Fine-motor subscores showed nominal positive associations with ODI in the left (ρ = 0.48, *p* = 0.021, uncorrected) and right amygdala (ρ = 0.51, *p* = 0.012, uncorrected). Gross motor subscores were nominally associated with NDI in the right thalamus (ρ = 0.58, *p* = 0.004, uncorrected), left thalamus (ρ = 0.42, *p* = 0.047, uncorrected), left caudate (ρ = 0.52, *p* = 0.011, uncorrected), and right caudate (ρ = 0.57, *p* = 0.033, uncorrected). None of these associations survived false discovery rate correction and should therefore be interpreted as exploratory.

**Discussion:**

These findings suggest subtle and predominantly exploratory associations between DGM microstructural properties and clinical measures in ALS. NODDI derived metrics, particularly ODI and NDI, may provide sensitive indices of subcortical microstructural variation, warranting further investigation in larger cohorts.

## Introduction

Amyotrophic lateral sclerosis (ALS) is a fatal neurodegenerative disorder characterized by progressive degeneration of upper and lower motor neurons, ultimately leading to paralysis, respiratory failure, and death (Hardiman et al., 2017). Traditionally known as a disease limited to the motor system, growing neuropathological and neuroimaging evidence now supports a broader, multisystem model of ALS that includes extensive cortical and subcortical involvement (Castelnovo et al., 2023). The subcortical gray matter, including the thalamus, basal ganglia, hippocampus, and amygdala, plays a central role in integrating motor and cognitive functions through its extensive connections with cortical and brainstem networks (Dieckmann et al., 2022; Bede et al., 2018). However, its contribution to the onset and progression of ALS, and how its degeneration relates to patients’ function/clinical decline, remains not entirely understood. Growing evidence from postmortem and neuroimaging studies indicates that these subcortical regions are particularly vulnerable in ALS, showing progressive atrophy that parallels cortical gray matter loss as the disease progresses (Castelnovo et al., 2023; Dieckmann et al., 2022; Bede et al., 2018).

Magnetic resonance imaging (MRI) has transformed our ability to study ALS in vivo by revealing both macrostructural atrophy and microstructural disorganization across the motor network. Diffusion tensor imaging (DTI) has identified white-matter degeneration in the corticospinal tracts, corpus callosum, and frontotemporal connections (Barritt et al., 2018; Kassubek et al., 2012). However, conventional DTI metrics, such as fractional anisotropy (FA) and mean diffusivity, are impacted by Gaussian diffusion assumptions and offer limited biological specificity, particularly in gray matter regions with complex axonal and dendritic architectures. To move beyond these limitations, advanced diffusion models have been developed to capture the microstructural composition of neural tissue with greater precision.

Among these approaches, neurite orientation dispersion and density imaging (NODDI) provides a biophysically grounded model of brain microstructure by estimating the neurite density index (NDI), orientation dispersion index (ODI), and free water fraction (FWF), which quantify neurite packing, geometric complexity, and extracellular water content, respectively (Zhang et al., 2012). NODDI has proven more sensitive than DTI in detecting subtle microstructural changes across neurodegenerative disorders, including Parkinson’s disease, multiple sclerosis, and Alzheimer’s disease (Kamagata et al., 2016; Grussu et al., 2017; Kamiya et al., 2020). In ALS, Broad et al. (2019) reported reductions in both NDI and ODI within the corticospinal tract and precentral gyrus, indicative of coexisting axonal loss and dendritic simplification. More recent studies have demonstrated widespread cortical and subcortical alterations in NODDI metrics, highlighting a link between microstructural disorganization and both motor and cognitive impairment (Xu et al., 2024; Xiao et al., 2024). Complementary diffusion frameworks, such as mean apparent propagator (MAP) MRI, have further identified thalamic and callosal abnormalities associated with disease severity (Chen et al., 2021). Collectively, these findings demonstrate that NODDI and related diffusion models provide a biologically meaningful window into the microstructural underpinnings of ALS.

Beyond the motor cortex, evidence from connectome-based modeling suggests that ALS pathology spreads through structural networks anchored in cortical and subcortical hubs (Meier et al., 2020). The thalamus and basal ganglia occupy key positions within these circuits, integrating motor, cognitive, and respiratory information. Degeneration of these subcortical nuclei may therefore contribute to both motor dysfunction and non-motor symptoms, reflecting downstream or trans-synaptic propagation of disease (Canna et al., 2021; Castelnovo et al., 2023; Pirozzi et al., 2025). However, despite their importance, the *in vivo* microstructural characterization of subcortical gray matter and its clinical relevance remain insufficiently defined.

In this study, we aimed to provide a detailed characterization of subcortical gray matter microstructure in ALS using NODDI, a diffusion-MRI model capable of disentangling neurite density, orientation dispersion, and extracellular free water. Using a region-of-interest approach encompassing the thalamus, caudate, putamen, pallidum, hippocampus, and amygdala, we investigated how subcortical neurite organization differs between patients with ALS and healthy controls, and how these microstructural alterations relate to clinical impairment. By focusing on deep gray matter (DGM) structures, regions often overlooked in diffusion-based ALS research, this study offers a novel perspective on the subcortical architecture of neurodegeneration. Our approach seeks to clarify the contribution of subcortical network disruption to ALS-related disability and to establish NODDI-derived microstructural indices as biologically specific and candidate imaging biomarkers for tracking disease progression and evaluating therapeutic interventions.

## Materials and methods

### Study population

Twenty-three patients (12 females, 11 males; mean age: 60.43 ± 13.18, range: 44–76 years) affected by definite, clinical, or laboratory-supported probable ALS according to the El-Escorial revised criteria (Brooks et al., 2000) and 24 healthy control (HC) subjects (11 females, 13 males; mean age: 59.67 ± 12.80; range: 27–83) consecutively underwent MRI acquisition at the “Advanced MRI Center” of the University of Campania “Luigi Vanvitelli” (Naples, Italy) from January 2023 to January 2024. HC were age-, and sex-matched with the enrolled ALS patients. Patient and control characteristics are reported in Table 1.

**TABLE 1:** Demographic and clinical measures of amyotrophic lateral sclerosis (ALS) patients.

Demographics	ALS	HC	*t*-test//χ2 (*p*-value)
Clinical features	(*n* = 23)	(*n* = 24)	ALS vs. HC
**Demographics**			
Age, years (range)	60.43 ± 13.18 (44–76)	59.67 ± 12.80 (27–83)	0.83 (0.41)
Sex, female (%)	12 (52.17%)	11 (45.83%)	0.19 (0.66)
**Clinical features**			
Disease duration, months (range)	12.30 ± 10.37 (3–48)	–	–
ALSFRS-R, total (range)	38.65 ± 5.97 (26–47)	–	–
ALSFRS-R, bulbar (range)	11.17 ± 1.47 (6–12)	–	–
ALSFRS-R, fine motor (range)	8.09 ± 2.91 (2–11)	–	–
ALSFRS-R, gross motor (range)	7.57 ± 3.33 (2–12)	–	–
ALSFRS-R, respiratory (range)	11.61 ± 30.94 (8–12)	–	–
UMN score (range)	7.87 ± 4.24 (0–14)	–	–

ALS, amyotrophic lateral sclerosis; HC, healthy controls; ALSFRS-R, ALS Functional Rating Scale-Revised; UMN, upper motor neuron; χ2, Chi-square test.

Amyotrophic lateral sclerosis patients met the following criteria: classic, bulbar, Lower Motor Neuron (LMN)- or Upper Motor Neuron-dominant (UMN-d) phenotypes (Chiò et al., 2011); disease onset not earlier than 36 months from enrollment; the onset age of 40 years or older. Clinical assessment of all ALS patients included the revised ALS Functional Rating Scale (ALSFRS-R) (Cedarbaum et al., 1999), with total and subdomains scores (bulbar, fine motor, gross motor, and respiratory) to quantify functional impairment across four domains; and an upper motor neuron (UMN) score to evaluate pyramidal dysfunction (Turner et al., 2004). Genetic testing was performed on all patients, including screening for *C9orf72* repeat expansions and mutations in *SOD1, TARDBP, and FUS/TLS* (as well as *SPAST* and *SPG7* in UMN-d cases). No pathogenic mutations were reported.

The study was conducted according to the Declaration of Helsinki and approved by the Ethics Committee of the University of Campania “Luigi Vanvitelli” (Protocol nr. 591/2018). Written informed consent was obtained from each participant before MRI acquisition.

### MRI protocol

Magnetic resonance imaging data were acquired using a 3T GE Discovery MR750 scanner equipped with a 32-channel head coil (Signa HDxt, GE Healthcare, Milwaukee, Wisconsin), following the specified protocol:

(1)    Anatomical 3D-T1-weighted (3D-T1w) images were obtained using a 3D inversion-recovery spoiled gradient-echo sequence (IR-SPGR, GE Healthcare) with the following parameters: echo time (TE) = 2.996 ms; readout repetition time (TR) = 6.9 ms; inversion time (TI) = 650 ms; flip angle = 9°; field of view (FoV) = 256 × 256 mm^2^; matrix = 256 × 256; slice thickness = 1 mm; acquisition time = 6.49 min.
(2)    Multishell Diffusion-Weighted MRI (DW-MRI) was acquired using a spin-echo echo planar imaging (EPI) sequence with three different b-values: 30 directions at b = 700 s/mm^2^, 30 directions at b = 1,000 s/mm^2^ and 64 diffusion directions at b = 2,000 s/mm^2^, along with 13 non-diffusion-weighted (b = 0) volumes. The following were additional acquisition parameters: TE = 75 ms; TR = 5,500 ms; FoV = 256 × 256 mm^2^; matrix = 128 × 128; slice thickness = 2 mm. An additional series with reversed phase-encoding direction was acquired for DW imaging distortion correction, consisting of nine volumes (3 b = 0 and 6 b = 2,000 s/mm^2^), using the same acquisition parameters as the multishell sequence. The total acquisition time was approximately 13 min.

### MRI data processing

All 3D-T1w anatomical images were processed using FreeSurfer (version 7.0) (Fischl, 2012) via the standard *recon-all* structural imaging pipeline, including skull stripping, intensity normalization and tissue segmentation according to the parcelation provided by the Destrieux atlas (Destrieux et al., 2010). The subcortical deep matter (DGM) regions of interest (ROIs) considered in this study were the left and right thalamus, caudate, putamen, pallidum, hippocampus and amygdala. Total intracranial volume (ICV) and volumetric measures for all ROIs were computed.

Multishell DW-MRI data were denoised and corrected for common acquisition-related artifacts. Denoising was performed using the *dwidenoise* tool from MRtrix3^1^, which removes noise-only principal components based on random matrix theory (Veraart et al., 2016; Cordero-Grande et al., 2019). Susceptibility-induced geometric distortions were corrected using the FSL (FMRIB Software Library^2^) tool *topup*, based on pairs of reverse phase-encoded *b* = 0 images. Brain extraction was performed using the FSL tool *bet.* Eddy-current–related distortions and subject motion were corrected using FSL *eddy*, incorporating the susceptibility field estimated by *topup* (Andersson and Sotiropoulos, 2016). Data quality was assessed at the subject level using the *eddy_quad* module from the quality control EDDY-QC framework in FSL (Bastiani et al., 2019) to verify that the signal-to-noise ratio (SNR) for all diffusion images was SNR ≥ 15 (Dell’Acqua and Tournier, 2019; Cirillo et al., 2025). Subsequently, a diffusion tensor model was fitted voxel-wise in the preprocessed images using the *dtifit* tool from FSL. Diffusion tensor fitting was restricted to the b = 0 and b = 1,000 s/mm^2^ shells, resulting in the computation of FA maps.

Furthermore, DW-MRI data were analyzed using the NODDI (Zhang et al., 2012) toolbox (version 1.0.4^3^) implemented in MATLAB (version R2019b, The MathWorks Inc., Natick, MA) to model the voxel-wise DW signal as the contribution of three microstructural compartments: (1) an intra-neurite compartment restricted diffusion within neurites, (2) an extra-neurite compartment representing hindered diffusion in the extracellular space, and (3) an isotropic compartment accounting for freely diffusing water, typically associated with cerebrospinal fluid or other free water components within the tissue (Zhang et al., 2012). The model provided three microstructural indices: NDI, ODI, and isotropic volume fraction (*fiso*), the latter used here as an estimate of the FWF (Palacios et al., 2020).

Finally, FA maps were co-registered from native diffusion space to each subject’s anatomical space using FMRIB’s Linear Registration Tool (FLIRT-FSL) (Jenkinson and Smith, 2001; Jenkinson et al., 2002). The corresponding subcortical DGM segmentations were then transformed into diffusion space by applying the inverse transformation. These concatenated registrations enabled the extraction of subject-specific mean values in ROIs from all NODDI-derived maps (NDI, ODI, FWF) within native diffusion space (hereinafter referred to as regional NODDI-derived metrics).

### Statistical analysis

Group matching between ALS patients and healthy controls (HC) was verified using the Wilcoxon rank-sum test for age and the chi-square test for sex.

Regional volumes and NODDI-derived metrics (NDI, ODI, FWF) were adjusted for the effect of age and sex using linear regression. For volumetric analyses of subcortical regions, ICV was additionally included as a covariate to account for inter-individual differences in head size. Between-group differences in adjusted regional volumes and NODDI-derived metrics were assessed using Wilcoxon rank-sum tests. Given the known heterogeneity of ALS and reports of asymmetric neurodegeneration (Yoganathan et al., 2025), left and right hemispheric measures were analyzed separately as distinct outcomes to allow detection of potential asymmetric patterns of subcortical involvement.

Within the ALS group, associations between regional NODDI-derived metrics and clinical measures (ALSFRS-R total and subdomain scores, and UMN score) were first assessed using Spearman’s rank correlation on age- and sex-adjusted NODDI-derived metrics. Then, for visualization and evaluation of trend robustness, significant correlations were further explored with both ordinary least squares (OLS) regression and robust regression (*robustfit*). Robust regression was used to account for potential outliers and high-leverage observations that might disproportionately influence standard least-squares regression, especially with relatively small sample sizes. It was implemented using iteratively reweighted least squares with Tukey’s bisquare weighting (tuning constant = 4.685), which reduces the influence of observations with large residuals rather than excluding them (Holland and Welsch, 1977; Huber, 1981). Coefficients of determination (R^2^) and the corresponding *p*-values for regression slopes were reported.

All statistical analyses were conducted in MATLAB (R2022b; The MathWorks, Natick, MA, United States). Statistical significance was set at *p* < 0.05. Multiple comparisons in group comparison and correlation analyses were corrected using the Benjamini–Hochberg false discovery rate (FDR) procedure (Benjamini and Hochberg, 1995).

## Results

**FIGURE 1:**
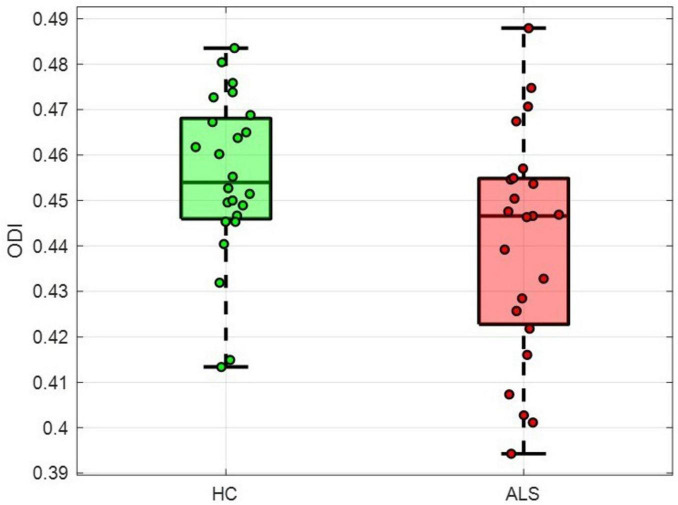
Boxplots of orientation dispersion index (ODI) values in the right putamen for amyotrophic lateral sclerosis (ALS) patients and healthy controls (HC).

No significant volumetric differences were observed between ALS patients and HCs in any of the DGM-ROIs (all *p* > 0.05, FDR-corrected). Among the NODDI-derived metrics, no group differences survived FDR correction (all *p* > 0.05, FDR-corrected). At the uncorrected level, the ODI in the right putamen was nominally lower in ALS patients (Figure 1) compared with HCs (z = −2.01, *p* = 0.044, uncorrected).

**FIGURE 2:**
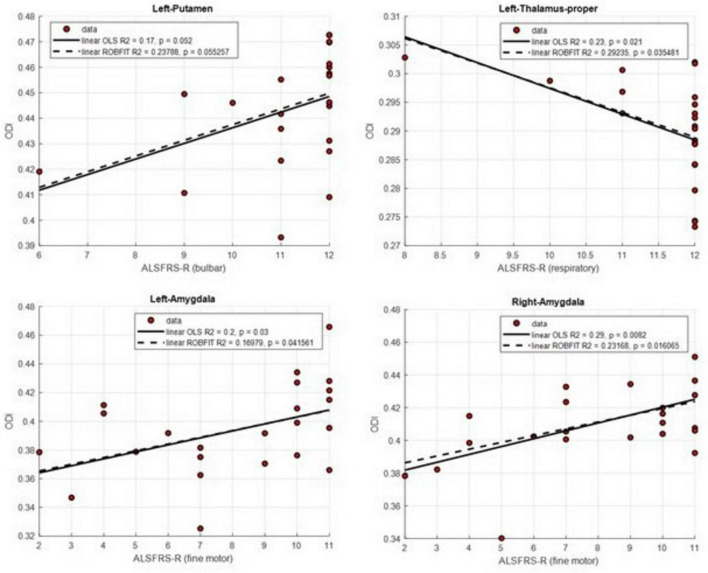
Scatterplots illustrating the significant relationships between ALS Functional Rating Scale–Revised (ALSFRS-R) subscores and mean orientation dispersion index (ODI) values in deep gray matter (DGM) regions. Linear trends were fitted using ordinary least squares (OLS, solid lines) and robust regression (dashed lines). The plots show: (top left) ALSFRS-R bulbar subscore vs. left putamen; (top right) ALSFRS-R respiratory subscore vs. left thalamus; (bottom left) ALSFRS-R fine motor subscore vs. left amygdala; and (bottom right) ALSFRS-R fine motor subscore vs. right amygdala.

Spearman correlation analyses were performed to investigate the associations between microstructural properties measured by NODDI-derived metrics (ODI, NDI, FWF) and clinical scores (ALSFRS-R total score and subscores, and UMN score) in ALS patients. For ODI, no correlation remained significant after FDR correction. However, several uncorrected significant associations emerged within DGM-ROIs. The ALSFRS-R bulbar subscore was found to be positively correlated with ODI in the left putamen (ρ = 0.50, *p* = 0.015, uncorrected), while the fine motor subscore showed positive correlations with ODI in both left (ρ = 0.48, *p* = 0.021, uncorrected) and right amygdala (ρ = 0.51, *p* = 0.012, uncorrected). Conversely, lower ALSFRS-R respiratory subscores were associated with higher ODI in the left thalamus (ρ = 0.57, *p* = 0.0047, uncorrected) (Figure 2). The positive association between ALSFRS-R fine motor subscore and ODI was supported in both the left (OLS: R^2^ = 0.20, *p* = 0.03; robust: R^2^ = 0.17, *p* = 0.041) and right amygdala (OLS: R^2^ = 0.29, *p* = 0.0082; robust: R^2^ = 0.23, *p* = 0.016), indicating consistent trends with minimal outlier influence. Similarly, a nominal inverse trend between respiratory function and ODI in the left thalamus was observed across regression models (OLS: R^2^ = 0.23, *p* = 0.021; robust: R^2^ = 0.29, *p* = 0.035), indicating consistency in direction but not robustness to multiple-comparisons correction. While for the left putamen, the positive trend observed between ALSFRS-R bulbar and ODI did not reach significance in either regression model (OLS: R^2^ = 0.17, *p* = 0.052; robust: R^2^ = 0.24, *p* = 0.055).

**FIGURE 3:**
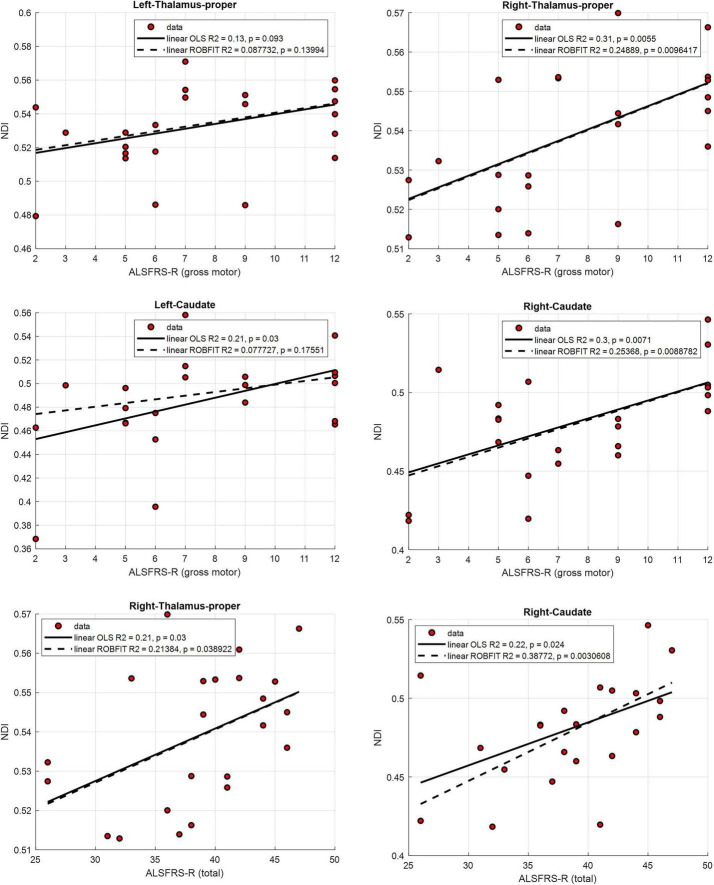
Scatterplots illustrating the relationships between ALS Functional Rating Scale–Revised (ALSFRS-R) scores and mean neurite density index (NDI) values in deep gray matter regions. Linear trends were fitted using ordinary least squares (OLS, solid lines) and robust regression (dashed lines). The plots show correlations between the ALSFRS-R gross motor subscore and NDI in the left (top left) and right thalamus (top right), as well as in the left (middle left) and right caudate (middle right). The bottom panels depict correlations between ALSFRS-R total score and NDI in the right thalamus (bottom left) and right caudate (bottom right). Significant positive associations were confirmed by both regression models for the right-sided regions.

For NDI, a significant positive correlation between the ALSFRS-R gross motor subscore and NDI in the right thalamus survived FDR correction (ρ = 0.58, *p* = 0.004, FDR-corrected *p* = 0.046). Other uncorrected positive correlations were observed between ALSFRS-R gross motor subscore and NDI in the left thalamus (ρ = 0.42, *p* = 0.047, uncorrected), left (ρ = 0.52, *p* = 0.011, uncorrected), and right caudate (ρ = 0.57, *p* = 0.033, uncorrected). Positive associations were also found between ALSFRS-R total score and NDI in the right thalamus (ρ = 0.46, *p* = 0.025, uncorrected) and right caudate (ρ = 0.48, *p* = 0.020, uncorrected) (Figure 3). Bilateral trends were observed between the ALSFRS-R gross motor subscore and NDI in the thalamic and caudate regions, although only the right-sided reached statistical significance in the regression. Specifically, both OLS and robust regressions supported the positive association between ALSFRS-R gross motor subscore and NDI in the right thalamus (OLS: R^2^ = 0.31, *p* = 0.0055; robust: R^2^ = 0.25, *p* = 0.0096) and right caudate (OLS: R^2^ = 0.30, *p* = 0.0071; robust: R^2^ = 0.25, *p* = 0.0089). In the left thalamus, a similar but weaker trend was observed (OLS: R^2^ = 0.13, *p* = 0.093; robust: R^2^ = 0.09, *p* = 0.14), while in the left caudate the relationship reached significance only in OLS regression (OLS: R^2^ = 0.21, *p* = 0.03; robust: R^2^ = 0.08, *p* = 0.18). Significant trends were also consistent for the associations between ALSFRS-R total score and NDI in the same right-sided DGM nuclei. The positive relationships were supported by both regression methods for the right thalamus (OLS: R^2^ = 0.22, *p* = 0.024; robust: R^2^ = 0.39, *p* = 0.0031) and right caudate (OLS: R^2^ = 0.21, *p* = 0.03; robust: R^2^ = 0.21, *p* = 0.039).

**FIGURE 4:**
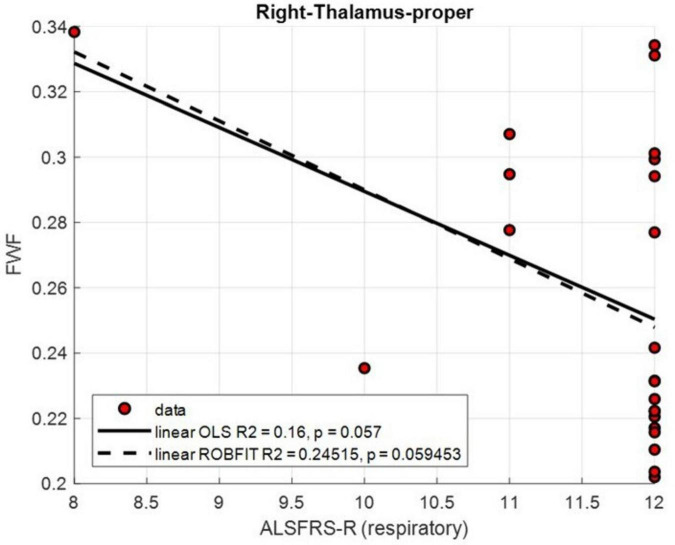
Scatterplots illustrating the relationships between ALS Functional Rating Scale–Revised (ALSFRS-R) respiratory subscore and free water fraction (FWF) values in the right thalamus. Linear trends were fitted using ordinary least squares (OLS, solid lines) and robust regression (dashed lines).

For FWF, no correlation survived FDR correction. At the uncorrected level, a significant negative correlation was found between the ALSFRS-R respiratory subscore and FWF in the right thalamus (ρ = −0.43, *p* = 0.039, uncorrected). The corresponding scatterplot (Figure 4) illustrates this inverse relationship. Although a negative trend was consistently observed, it did not reach statistical significance in either regression model (OLS: R^2^ = 0.16, *p* = 0.057; robust: R^2^ = 0.25, *p* = 0.059).

## Discussion

This study explored the relationship between DGM microstructure and clinical disability in ALS, despite the absence of robust between-group differences at the volumetric or nODDI-derived metric levels. although no group comparisons survived multiple-comparison correction, several biologically plausible exploratory associations emerged between DGM microstructural indices and clinical disability, suggesting that subtle subcortical microstructural variability may relate to clinical heterogeneity in ALS. these observations are consistent with the concept of ALS as a multisystem disorder involving both cortical and subcortical circuits (Castelnovo et al., 2023; Dieckmann et al., 2022) and underscore the potential value of advanced diffusion models for detecting microstructural alterations that may not be captured by conventional diffusion imaging approaches (Broad et al., 2019; Kamiya et al., 2020).

From a broader conceptual perspective, the present results align with the expanding literature demonstrating the sensitivity of NODDI-derived metrics to microstructural alterations in ALS and other neurodegenerative disorders. Recent ALS studies have reported thalamic and thalamo-cortical microstructural abnormalities, with neurite density and orientation metrics exhibiting greater sensitivity and stronger clinical associations than traditional diffusion tensor imaging measures (Cao et al., 2024). Converging evidence from Alzheimer’s and Parkinson’s disease further indicates that NODDI-derived metrics are sensitive to early gray and white matter microstructural variability within subcortical and limbic regions and relate to clinical and cognitive phenotypes, even in the absence of overt macroscopic atrophy (Yu et al., 2024; Parker et al., 2025; Lavrova et al., 2025; Zhu et al., 2025). Taken together, these studies provide an appropriate contextual framework for interpreting the predominantly exploratory associations observed in the present study.

In light of this framework, a nominal association between thalamic ODI and respiratory ALSFRS-R subscores was identified. However, as this relationship did not survive correction for multiple comparisons, it should be interpreted strictly as exploratory and not as evidence of a specific mechanistic link. Although increased ODI has been associated with altered neurite orientation, dendritic remodeling, or glial-related microstructural changes (Yi et al., 2019). The biological interpretation of ODI in gray matter remains inherently non-specific in cross-sectional *in vivo* studies. The thalamus plays an established role in integrating descending motor signals with brainstem respiratory circuits, and its vulnerability in ALS has been reported in both volumetric and advanced diffusion imaging studies (Chen et al., 2021; Dieckmann et al., 2022). According to contemporary network-based models of ALS, subcortical structures such as the thalamus are thought to participate in broader structurally and functionally connected disease-spreading networks rather than acting as primary pathological hubs (Meier et al., 2020; Chipika et al., 2020b).

Importantly, ODI and NDI in gray matter do not map uniquely onto neurite organization or density and may instead reflect a composite of microstructural features, including dendritic complexity, glial contributions, extracellular space, and partial volume effects, particularly in cross-sectional *in vivo* studies (Niu et al., 2025). While higher ODI has often been interpreted as reflecting increased neurite disorganization, altered dendritic architecture, or glial-related microstructural changes, alternative explanations related to compensatory or adaptive remodeling cannot be excluded. Similar interpretational challenges have been reported in other neurodegenerative disorders. For example, in early Parkinson’s disease, increased ODI in subcortical and cortical regions has been variably attributed to compensatory dendritic remodeling, synaptic reorganization, or early microstructural instability preceding overt neurodegeneration (Kamagata et al., 2016; Grussu et al., 2017). These findings suggest that ODI changes may reflect a dynamic balance between degenerative and compensatory processes rather than a unidirectional marker of structural breakdown (Kamiya et al., 2020). In the absence of longitudinal data, it is therefore not possible to disentangle whether increased ODI in ALS reflects early compensatory responses within subcortical networks or emerging microstructural disorganization. Longitudinal and multimodal imaging studies will be essential to clarify the temporal trajectory and biological significance of ODI alterations across disease stages.

Along these lines, additional nominal associations were observed between ODI in the amygdala and putamen and fine-motor and bulbar ALSFRS-R subscores, suggesting a potential functional relevance of DGM microstructural variability. Although these findings did not survive correction for multiple comparisons, they remain anatomically plausible. Previous work has demonstrated selective involvement of amygdalar nuclei in ALS, supporting the contribution of limbic structures to disease-related clinical features (Chipika et al., 2020a). Independent structural connectivity studies in healthy humans have further demonstrated amygdala connections with premotor and motor-related cortical regions, providing anatomical support for potential emotional–motor interactions without implying disease-specific mechanistic pathways (Toschi et al., 2017). The putamen, in turn, is a key component of basal ganglia loops supporting movement initiation and coordination, and basal ganglia involvement has been consistently reported in ALS (Castelnovo et al., 2023). In this context, the observed ODI associations may reflect subtle microstructural variability in these nuclei that relates to functional decline even in the absence of overt atrophy. This interpretation is consistent with reports from other neurodegenerative disorders, where NODDI has revealed early or subtle changes in gray matter (Grussu et al., 2017; Kamagata et al., 2016).

The strongest statistical finding in this study was the positive association between NDI in the right thalamus and gross-motor ALSFRS-R subscores, which was the only relationship to survive correction for multiple comparisons. However, the remaining positive correlations between NDI and clinical measures, including those involving the thalamus and caudate, as well as total ALSFRS-R scores, did not survive correction and should therefore be interpreted as exploratory. Higher NDI generally indicates greater axonal/dendritic packing; in this context, patients with better preserved motor function tended to show higher NDI in the thalamus and caudate. These observations align with previous reports of widespread NDI reductions in ALS (Xu et al., 2024; Xiao et al., 2024) and are consistent with the notion that reduced neurite density parallels clinical decline. Notably, consistency across both OLS and robust regression analyses suggests limited influence of extreme outliers (Holland and Welsch, 1977; Huber, 1981).

In addition, a nominal negative association between thalamic FWF and respiratory function was also observed, suggesting a possible increase in extracellular free water in ALS patients with poorer respiratory capacity. However, given the non-specific nature of FWF and the fact that this association did not withstand correction for multiple comparisons, this finding should be considered exploratory.

The absence of significant group differences in DGM volumes or NODDI metrics may reflect several factors. First, DGM nuclei may undergo relatively subtle microstructural alterations compared with the prominent corticospinal and frontotemporal white matter changes typically reported in ALS (Barritt et al., 2018; Kassubek et al., 2012). Second, NODDI indices may capture heterogeneity within ALS phenotypes, ranging from limb to bulbar to UMN-dominant presentations, resulting in inter-individual variability that reduces statistical power, obscuring group-level effects. Notably, the correlation-based findings indicate that clinical relevance may emerge even in the absence of stark between-group differences, underscoring the sensitivity of NODDI to continuous variation across disease severity.

Another possible interpretation is that preserved or even elevated NDI/ODI in specific DGM regions may reflect compensatory remodeling rather than pure degeneration. Similar compensatory subcortical mechanisms have been postulated in other neurodegenerative disorders, including Parkinson’s disease and Huntington’s disease, based on NODDI findings (Grussu et al., 2017; Kamagata et al., 2016). In ALS, the basal ganglia and thalamus may attempt to adaptively support motor output when corticospinal circuits deteriorate (Castelnovo et al., 2023). Accordingly, associations between microstructural complexity and functional scores may reflect dynamic compensation, rather than structural loss. However, future longitudinal studies are required to disentangle compensatory versus degenerative trajectories and to confirm whether increased NDI/ODI reflects an adaptive response. While not yet suitable as standalone diagnostic markers, NODDI-derived metrics may complement traditional imaging by offering biologically specific indices of neurite integrity and organization. Given the pressing need for sensitive biomarkers capable of tracking disease progression and therapeutic response (Barritt et al., 2018), DGM-focused NODDI metrics may provide an important contribution, especially in multidomain ALS phenotypes.

Several limitations should be acknowledged when interpreting the present findings in this broader context. The relatively small sample size limited statistical power to detect group-level differences across multiple ROIs, likely contributing to the absence of significant effects after correction for multiple comparisons. In addition, uncorrected correlation analyses are inherently more susceptible to false-positive findings in the setting of extensive multiple testing. Although robust regression analyses reduced sensitivity to outlier influence, multiple-comparison correction eliminated most associations, warranting cautious interpretation of the remaining uncorrected results. Furthermore, the cross-sectional design precludes characterization of longitudinal microstructural change, thereby limiting inferences regarding disease progression. Accordingly, the generalizability of the present findings to the broader ALS population remains constrained.

Despite these limitations, the observed associations, particularly those involving thalamic ODI and NDI, suggest that DGM microstructure may relate to clinically relevant aspects of ALS-related disability. However, these findings should be regarded as exploratory and hypothesis-generating, requiring confirmation in future hypothesis-driven studies. Such investigations should also account for hemispheric effects and potential interactions on NODDI-derived microstructural metrics, which may offer greater microstructural specificity and reveal subcortical-level patterns potentially overlooked in the present study. Future studies with larger, phenotypically stratified cohorts, longitudinal NODDI acquisitions, and comprehensive assessment of non-motor domains, including cognition, sleep–wake regulation, appetite, and energy metabolism, will be essential to determine whether DGM microstructural alterations, mainly in the thalamus, basal ganglia, hippocampus, and amygdala, are also associated with cognitive and other non-motor manifestations of ALS.

## Conclusion

In this study, we applied neurite orientation dispersion and density imaging to investigate DGM microstructure and its relationship with clinical disability in ALS. By focusing on subcortical regions that have received relatively limited attention in ALS imaging research, we identified predominantly exploratory associations between NODDI-derived microstructural metrics and respiratory, gross-motor, bulbar, and fine-motor impairments. Although none of these associations survived correction for multiple comparisons, the observed patterns suggest that subcortical microstructural variability may contribute to clinical heterogeneity in ALS and highlight changes that may not be captured by conventional structural MRI. While these findings require confirmation in larger and longitudinal cohorts, they support the potential value of advanced diffusion models for probing subcortical involvement in ALS and for informing future longitudinal and therapeutic studies aimed at better characterizing clinically relevant aspects of disease progression.

## Data Availability

The raw data supporting the conclusions of this article will be made available by the authors, without undue reservation.

## Footnotes

1
https://mrtrix.readthedocs.io/en/dev/reference/commands/dwidenoise.html

2
https://fsl.fmrib.ox.ac.uk

3
http://www.nitrc.org/projects/noddi_toolbox
